# Integrin factor (FAM27E3), as a metastatic marker of papillary thyroid carcinoma, through the p53 signaling pathway promoting lymph node metastasis

**DOI:** 10.3389/fgene.2025.1593553

**Published:** 2025-07-30

**Authors:** Dong Zhang, Kai-Fang Xiang, Chen-Chen Hong, Xiao-Ping Bao, Yan Wu, Rui Wu

**Affiliations:** ^1^ Department of General Surgery, Kong Jiang Hosptal, Shanghai, China; ^2^ Department of Thyroid and Breast Surgery, Geriatric Hospital Affiliated with Wuhan University of Science and Technology, Wuhan, Hubei, China; ^3^ Oncology Department, The Sixth Hospital of Wuhan, Affiliated Hospital of Jianghan University, Wuhan, Hubei, China

**Keywords:** FAM27E3, PTC, lymph node metastasis, p53, macrophage

## Abstract

**Background:**

Papillary thyroid carcinoma (PTC) has a lymph node metastasis rate of 20%–90%. This study aims to explore the role and regulatory mechanism of the FAM27E3 in PTC lymph node metastasis.

**Method:**

The seven thyroid cancer (THCA) datasets from the Gene Expression Omnibus (GEO) were combined as the training group, while the PTC dataset served as the testing group. Venn analysis identified overlapping genes through consistent cluster analysis and weighted gene co-expression network analysis (WGCNA). We used the Gene Ontology (GO) and Kyoto Encyclopedia of Genes and Genomes (KEGG) databases to perform gene set enrichment analysis (GSEA) to explore the enriched terms. Least absolute shrinkage and selection operator (LASSO) analysis was applied to the overlapping genes to identify key influence factors. Finally, FAM27E3 was used for score analysis (epithelial–mesenchymal transition (EMT), angiogenesis, and mRNAsi scores). Immune cell infiltration was assessed using the CIBERSORT algorithm. CCK8, colony formation, Transwell assays, Western blotting, and RT-PCR were employed to evaluate cell viability, invasion, migration, and gene/protein expression.

**Results:**

The clustering method distinguished the samples into two subtypes. We obtained the mRNA expression levels of 128 overlapping genes from the tumor and C1 modules of the GSE60542 dataset. FAM27E3 was identified as a hub gene associated with PTC lymph node metastasis and had a significantly positive correlation with EMT_score. The MRNAsi_score was increased in the FAM27E3 high-expression group. A high expression of FAM27E3 indicated a reduction in the overall macrophage levels. FAM27E3 exhibited high expression in PTC cell lines, and its downregulation suppressed cell proliferation, migration, and invasion. FAM27E3 promoted the lymph node metastasis of PTC, which was associated with the p53 signaling pathway.

**Conclusion:**

FAM27E3, as a marker for PTC lymph node metastasis, promotes PTC lymph node metastasis related to the p53 signaling pathway.

## 1 Introduction

Thyroid cancer (THCA) is the most prevalent endocrine tumor, and its global incidence continues to rise annually (Chen et al., 2023). According to the latest global cancer statistics by the World Health Organization (WHO) in 2023 (Wang et al., 2023), newly diagnosed cases of THCA have increased to seventh place among female cancer cases. THCA encompasses differentiated thyroid cancer (DTC) and medullary thyroid cancer (MTC). DTC originates from the follicular epithelial cells of the thyroid and includes papillary thyroid carcinoma (PTC), follicular thyroid carcinoma (FTC), and Hurthle cell carcinoma (HTC). PTC accounts for approximately 90% of all THCA. It is characterized as a slow-growing tumor with a favorable prognosis that tends to metastasize to lymph nodes in early stages (with a lymph node metastasis rate ranging from about 20% to 90%) (Evans, 1987; Min et al., 2021). Although PTC has a low mortality rate, some patients may present local or distant metastases at the initial examination, leading to a significant psychological burden and physical trauma for patients and their families. Therefore, it is imperative to adhere to the principle of “early detection, early diagnosis, and early treatment” for timely intervention in PTC. Identifying patients with PTC who are prone to experience local or distant metastasis in the early stages is crucial for doctors when determining surgical timing and formulating treatment plans. Understanding the mechanisms and pathways through which tumor cells metastasize and proliferate will greatly aid in enhancing control measures against PTC. The occurrence and spread of THCA are positively associated with high levels of integrin expression (Davis et al., 2020). Therefore, integrin ligand expression levels may be used to predict the occurrence or metastasis of THCA.

Integrins are a class of heterodimeric proteins located on the cell surface that serve as receptors for binding to the extracellular matrix (ECM) and play a crucial role in transmitting signals from the extracellular environment into the cell, thereby influencing signaling pathways associated with tumor cell survival, proliferation, and migration (Campbell and Humphries, 2011; Slack et al., 2022). Integrin α5β1 exhibits high expression in PTC but low expression in MTC, making it a valuable clinical diagnostic and prognostic marker for PTC (Karmakar and Mukherjee, 2003). LIMD2 is a pivotal protein driving tumor metastasis, responsible for facilitating signal communication between the cytoskeleton and the nucleus of cells. It can bind to integrin-linked kinase (ILK), enhancing ILK activity and regulating tumor cell movement and metastasis (Peng et al., 2014). Given its high expression in metastatic lesions of PTC, LIMD2 can be utilized to determine the spread of PTC (Pinheiro Dos Santos et al., 2018). Our group has developed and validated a novel risk model based on seven integrin-related gene signatures (FAM27E3, FIGN, GSTM4, BEX5, RBPM52, PHF13, and DCSTAMP) using data from the Cancer Genome Atlas (TCGA) database (Zhang et al., 2023). Our risk model demonstrates superior predictive capabilities in THCA with fewer genes than previous models. Additionally, most of the gene signatures in this model have the potential to serve as biomarkers for THCA. Therefore, the application of this model holds significance for further experimentation and treatment of THCA.

This study aims to analyze the relevant THCA datasets in the GEO database and identify the key integrin genes associated with PTC lymph node metastasis within the aforementioned risk model, with the objective of discovering novel biomarkers for PTC lymph node metastasis and conducting initial exploration to clarify the molecular mechanisms.

## 2 Materials and methods

### 2.1 Data downloading and processing

The data of GSE165706 (n = 21), GSE129562 (n = 16), GSE82208 (n = 52), GSE76039 (n = 37), GSE65144 (n = 25), GSE65074 (n = 38), GSE53157 (n = 27), GSE60542 (n = 92), and its corresponding platform files GPL570, GPL10558, and GPL13667 were acquired from the GEO database (https://www.ncbi.nlm.nih.gov/geo/). Then, the probe matrix of GSE165706, GSE129562, GSE82208, GSE76039, GSE65144, GSE65074, GSE53157, and GSE60542 was transformed into a gene matrix by using the Perl language. The data of GSE165706, GSE129562, GSE82208, GSE76039, GSE65144, GSE65074, and GSE53157 were combined and standardized and served as the training group using the “limma” and “sva” packages of R language software 4.2.0, while the data of GSE60542 served as the testing group due to its large sample size. In addition, the Cancer Genome Atlas (TCGA)-THCA dataset was used to explore the clinical value of key genes in predicting lymph node metastasis.

### 2.2 Cluster analysis of the combined dataset

Consensus clustering, a method to identify molecular subtypes based on an approximate number of clusters, was utilized to discover tumor subgroups by the k-means method. The maximum number of categories to be evaluated is 10, and the number of iterations for each k is 50. The Euclidean distance is selected as the clustering distance. Based on the combined dataset (GSE165706, GSE129562, GSE82208, GSE76039, GSE65144, GSE65074, and GSE53157), the number of clusters was calculated by the consensus clustering algorithm using the “ConsensuClusterPlus” R package. Graphical results included a heatmap of the consensus clustering, consensus cumulative distribution function (CDF) plots, and delta area plots. Meanwhile, principal component analysis (PCA) was used to detect whether the expression levels of these genes could distinguish between the samples from the different modules.

### 2.3 Identification of co-expression genes

Weighted gene co-expression network analysis (WGCNA) is an algorithm to cluster genes into different modules and uncover the relationships between modules and disease traits. To comprehensively investigate the genetic mechanisms involved in the pathogenesis of disease, a co-expression network was constructed by the “WGCNA” package in R. The co-expression network was built using the genes with the top 25% variance from the GSE96804 dataset. The dynamic cutting tree method was adopted to merge modules with a threshold of 0.25. Other criteria were used to construct the co-expression network: soft threshold power (β) based on the scale-free topology criterion (an independence index of *R* ^2^ = 0.85) using the pick soft threshold function; minimum genes of each module = 30. Pearson correlation analysis was adopted to reveal the potential correlations between modules and tumor, cluster (C) 1, and C2.

### 2.4 Acquisition of overlapped genes, GO, and KEGG analysis

Based on the three significant module genes, Venn analysis obtained 128 overlapping genes between the module tumor and module C1. The overlapping genes were included in GO and KEGG enrichment analysis using the “clusterProfiler” package.

### 2.5 Identification of the lymph node metastasis-related biomarkers of PTC

We obtained the mRNA expression levels of 128 overlapping genes from the GSE60542 dataset. Based on the presence of lymph node metastasis and vascular invasion in PTC patients, the least absolute shrinkage and selection operator (LASSO) algorithm was applied to identify the important influencing factors. The top 20 important influencing factors for both events and seven integrin-related gene signatures were included in Venn analysis to obtain PTC metastasis-related biomarkers.

### 2.6 Score method

#### 2.6.1 Angiogenesis score

The angiogenesis score for each sample in the present study was calculated using the “GSVA” R package. We then explored the correlation between angiogenesis score and FAM27E3 expression.

#### 2.6.2 EMT score

EMT score based on the 76-gene epithelial–mesenchymal transition (EMT) signatures (76 G) method was calculated as previously described for each tumor tissue sample derived from the TCGA cohort (Muralidharan et al., 2022).

#### 2.6.3 MRNAsi score

The MRNAsi score was calculated by the gene expression profiles of normal pluripotent stem cells (PSCs), including PSCs and embryonic stem cells (ESCs), which were collected by the Progenitor Cell Biology Consortium (PCMC, https://progenitorcells.org/). First, the 78 × 8087 stemness-related matrix for 78 stem cell samples and 8087 protein-coding genes was obtained, and the expression data were centered by the mean. Second, we acquired the stemness signature by the OCLR algorithm using the glmnet R package, which is a machine learning algorithm used to extract transcriptomic and epigenetic feature sets derived from nontrans formed pluripotent stem cells and their differentiated progeny. Third, the Spearman correlations between the weight vectors of the stemness signature and mRNA expression were determined (Huang et al., 2023). Finally, we obtained the mRNAsi score for each PTC sample.

### 2.7 Immune cell infiltration analysis

The R software’s “CIBERSORT” package was used to assess the level of immune cell infiltration based on 22 immune cell types of the “LM22” document (https://cibersort.stanford.edu/index.php). The results were filtered using the screening criteria: P-value <0.05. Depending on the results obtained by the immuno-infiltration assays, the differential expressions of 22 immune infiltrating cells between the two groups were visualized using violin plots. The correlation analysis of 22 infiltrating immune cell types was visualized by the R software’s “corrplot” package.

### 2.8 GSEA analysis of FAM27E3

Gene set enrichment analysis (GSEA) software (version 4.1.0) was used to compare the biological processes that were significantly different between the FAM27E3 low- and high-expression groups.

### 2.9 Cell culture

The Nthy-ori-3-1 normal thyroid cell line and the PTC cell lines IHH4 and TPC-1 were procured from Wuhan Punosai Life Science Co., Ltd., China. These cell lines were maintained in RPMI-164 basal medium (Gibco) supplemented with 10% fetal bovine serum (FBS, Gibco), 1% penicillin-streptomycin (100 U/mL penicillin, 100 μg/mL streptomycin, HyClone), and 25 μg/mL amphotericin B (Sigma-Aldrich). Cultures were routinely subcultured in a 37°C, 5% CO_2_ incubator.

### 2.10 ELISA assay for FAM27E3 level in PTC cell lines

The protein levels of FAM27E3 in Nthy-ori-3-1 (normal thyroid), IHH4, and TPC-1 (THCA) cells were quantified using a human-specific FAM27E3 ELISA kit (Catalog No. JN132560X, Shanghai Jinin Industrial Co., Ltd.). Cells were cultured to 80%–90% confluence in RPMI-1640 medium supplemented with 10% FBS and lysed in RIPA buffer containing protease inhibitors. Lysates were centrifuged at 12,000 × g for 15 min at 4°C, and supernatants were collected for analysis. Standards and samples (100 μL/well) were added to pre-coated plates in triplicate, followed by incubation with biotinylated detection antibody (1:200 dilution) and streptavidin–HRP conjugate (1:1000) according to the manufacturer’s protocol. After TMB substrate incubation (15 min, dark), reactions were terminated with 2M H_2_SO_4_, and absorbance was measured at 450 nm (reference: 570 nm) using a microplate reader (BioTek Synergy H1). FAM27E3 concentrations were calculated from the standard curve (0–100 ng/mL).

### 2.11 SiRNA transfection

SiRNA sequences for FAM27E3 and scrambled siRNA were purchased from Wuhan GeneCreate Biological Engineering Co., LTD (China). SiRNA was transfected into cells using Lipofectamine™ RNAiMAX (Thermofisher). The target sequences for siRNA were: scrambled siRNA, UUC​UCC​GAA​CGU​GUC​ACG​UTT; si-FAM27E3#1, UUA​UUA​UCA​ACA​CUG​UCC​CTT; si-FAM27E3#2, GGA​UUC​CAG​GUU​UAA​GAU​ATT.

### 2.12 Vector-p53 transfection

Vectors expressing p53 were constructed using the pcDNA3.1 vector (Genecreate Bioengineering Co., Ltd., Wuhan, China) as a backbone. TPC-1 cells were harvested at 75%–85% confluence and counted, followed by transfection of either 10 nM or 20 nM of the vector (referred to as “vector-p53 concentration (conc.) 1” and “vector-p53 conc.2”) into 10^6^ cells utilizing Lipofectamine 2000 (Thermo Fisher, San Jose, CA, USA). Cells transfected with empty vectors served as negative control cells (referred to as “vector”). All subsequent experiments were conducted using cells harvested at 48 h post-transfection.

### 2.13 RNA extraction and quantitative real-time polymerase chain reaction (qRT-PCR)

The total RNA from glomerular mesangial cells was extracted by TRIzol reagent (Tiangen Biotech, Beijing, China), and RNA concentration was measured using NanoDrop One/OneC (Thermo Scientific, USA). Subsequently, reverse transcription of RNA into cDNA was executed with HiScript^®^ II Q RT SuperMix for qPCR (Vazyme, Nanjing, China) according to the standard instructions. A LightCycler^®^ 96 Instrument (Roche, Switzerland) was used for qRT-PCR to detect mRNA expression. All the primers were synthesized by Wuhan GeneCreate Biological Engineering Co., LTD. The relative expression level of mRNA was calculated by the 2^−ΔΔCT^ method, and three replicate experiments were performed. Primers are shown in Table 1.

**TABLE 1:** Primers used for RT-PCR.

Gene	Species	Primer sequence (5′ to 3′)
FAM27E3	Human	Forward:TGATAGGAGACGGGGAGGC
		Reverse:ATGGGATGAGATGGGCAGG
E-cadherin	Human	Forward:CCTGGCCTCAGAAGACAGAA
		Reverse:TGGCCAGTGATGCTGTAGAA
CD206	Human	Forward:TGGGTGTCCGAATCTCAG
		Reverse:TGGCATTGCCTAGTAGCG
CD204	Human	Forward:GCACTGATTGCCCTTTAC
		Reverse:TTCCCGTGAGACTTTGAG
CD163	Human	Forward:AGTTGCCCTTTCTACCCC
		Reverse:CGACCTCCTCCATTTACC
GAPDH	Human	Forward:GGAGCGAGATCCCTCCAAAAT
		Reverse:GGCTGTTGTCATACTTCTCATGG

### 2.14 CCK-8 assay

The cell viability of transfected cells was assessed using the Cell Counting Kit-8 (CCK-8; Beyotime, Shanghai, China). Cells were seeded into 96-well plates at about 3000 cells per well. A 10-μL aliquot of CCK-8 reagent was added to each well. After incubation at 37°C for an additional 2 h, cell proliferation was determined by examining the absorbance at a wavelength of 450 nm using a microplate reader (Bio-Rad, USA).

### 2.15 Colony formation assay

Cells were seeded into 6-well plates at a seeding density of 1 × 10^3^ cells/well and were then transfected with siRNA or si-NC. Cells were cultured for 14 days and then fixed with methanol and stained with crystal violet. Colonies (>0.1 mm in diameter) were counted under a light microscope upon washing.

### 2.16 Transwell migration and invasion experiments

A Transwell chamber (24-well, 8.0-μm pore membranes (Corning USA)) was used according to the manufacturer’s protocol. Approximately 1 × 10^5^ cells per well were seeded in the upper chamber in 100 μL of serum-free medium, and 600 μL of complete medium was added to the lower chamber as a chemoattractant at the same time. After incubation for 24 h at 37°C, the cells remaining at the upper surface of the membrane were removed with cotton swabs. The cells on the lower surface of the membrane are the migrated cells. After fixation with 4% paraformaldehyde and staining with 0.1% crystal violet solution, the cells that passed through the filter were photographed using an inverted fluorescence microscope.

The Transwell invasion assay was carried out as described above, except that 100 μL of 1:8 DMEM-diluted Matrigel (BD, USA) was added to each well at 37°C for 6 h before the cells were seeded onto the membrane, followed by incubating for 48 h.

### 2.17 Western blot analysis

Total proteins were extracted from tissues and cells with RIPA buffer (Beyotime, China). Protein concentrations of whole extracts were measured using a BCA protein assay kit (Beyotime, China). Approximately 40 μg protein extract from each sample was separated using an SDS–polyacrylamide gel and transferred onto the PVDF membrane (Millipore, USA). The membranes were blocked with 5% skimmed milk and incubated with rabbit anti-E-cadherin (1:800; Beyotime, Shanghai, China), anti-p53 (1:1000; Beyotime), anti-phosphorylation-p53 (1:500; CST), and mouse antibody against GAPDH (1:2000; Beyotime) overnight at 4°C, followed by incubation for 1 h with horseradish peroxidase (HRP)-conjugated secondary antibodies (1:10,000; Beyotime). After extensive washing in PBST, the expression levels of the proteins were detected by Quantity One software (Bio-Rad Laboratories, USA) using the ECL-chemiluminescent kit.

### 2.18 Statistical analysis

The data were analyzed by SPSS (version 22.0; SPSS Science, Chicago, IL). Experiments were repeated at least three times in duplicate, and all data were reported as mean ± SD. Data were examined using Student’s unpaired t-test or one-way analysis of variance (ANOVA). FAM27E3 expression was divided into high- and low-expression groups according to the median (FAM27E3 median = 7.24). P < 0.05 was considered significant.

## 3 Results

### 3.1 Identifying THCA-associated molecular subgroups

**FIGURE 1:**
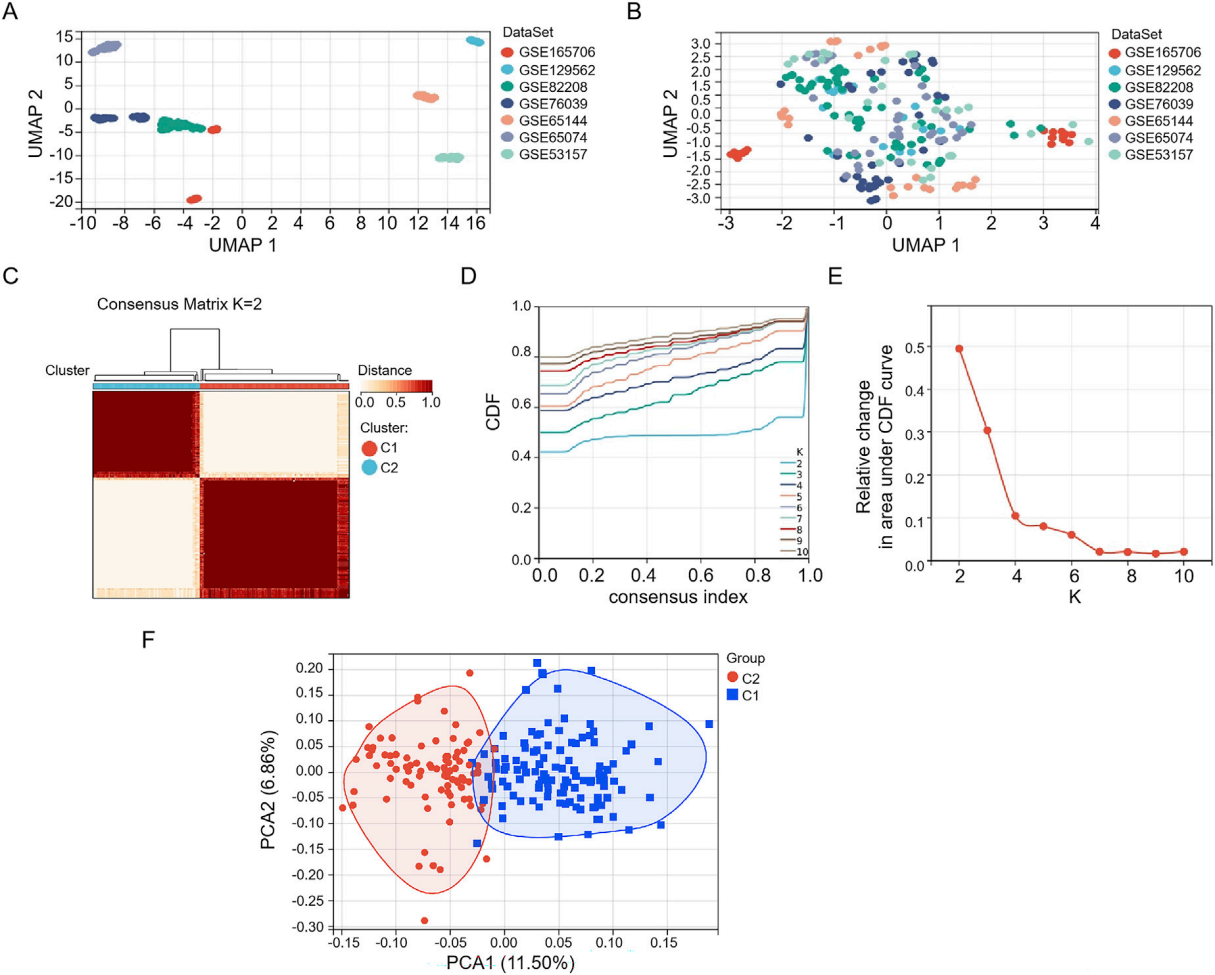
Consistent cluster analysis of the merged dataset. Seven GEO datasets **(A)** before and **(B)** after merging. **(C)** The 216 samples in the merged dataset were grouped into two clusters according to the consensus clustering matrix (k = 2). **(D)** Consensus among clusters for each category number k. **(E)** Delta area curves for consensus clustering indicating the relative change in area under the cumulative distribution function (CDF) curve for each category number k compared to k-1. The horizontal axis represents the category number k, and the vertical axis represents the relative change in area under the CDF curve. **(F)** PCA depiction of the distribution for clusters.

We first constructed a THCA-related merged dataset, including GSE165706, GSE129562, GSE82208, GSE76039, GSE65144, GSE65074, and GSE53157, which was set as a training set (Figures 1A,B). We performed a consensus clustering analysis with all 216 samples in the merged dataset. By increasing the clustering variable (k) from 2 to 10, we found that when k = 2, the intragroup correlations were the highest and the intergroup correlations were low, indicating that the 216 THCA patients could be well divided into two clusters (Figures 1C–E). Through PCA, we found that the clustering method effectively distinguished the samples into two subtypes (Figure 1F).

### 3.2 Identification of hub genes in modules related to THCA

**FIGURE 2:**
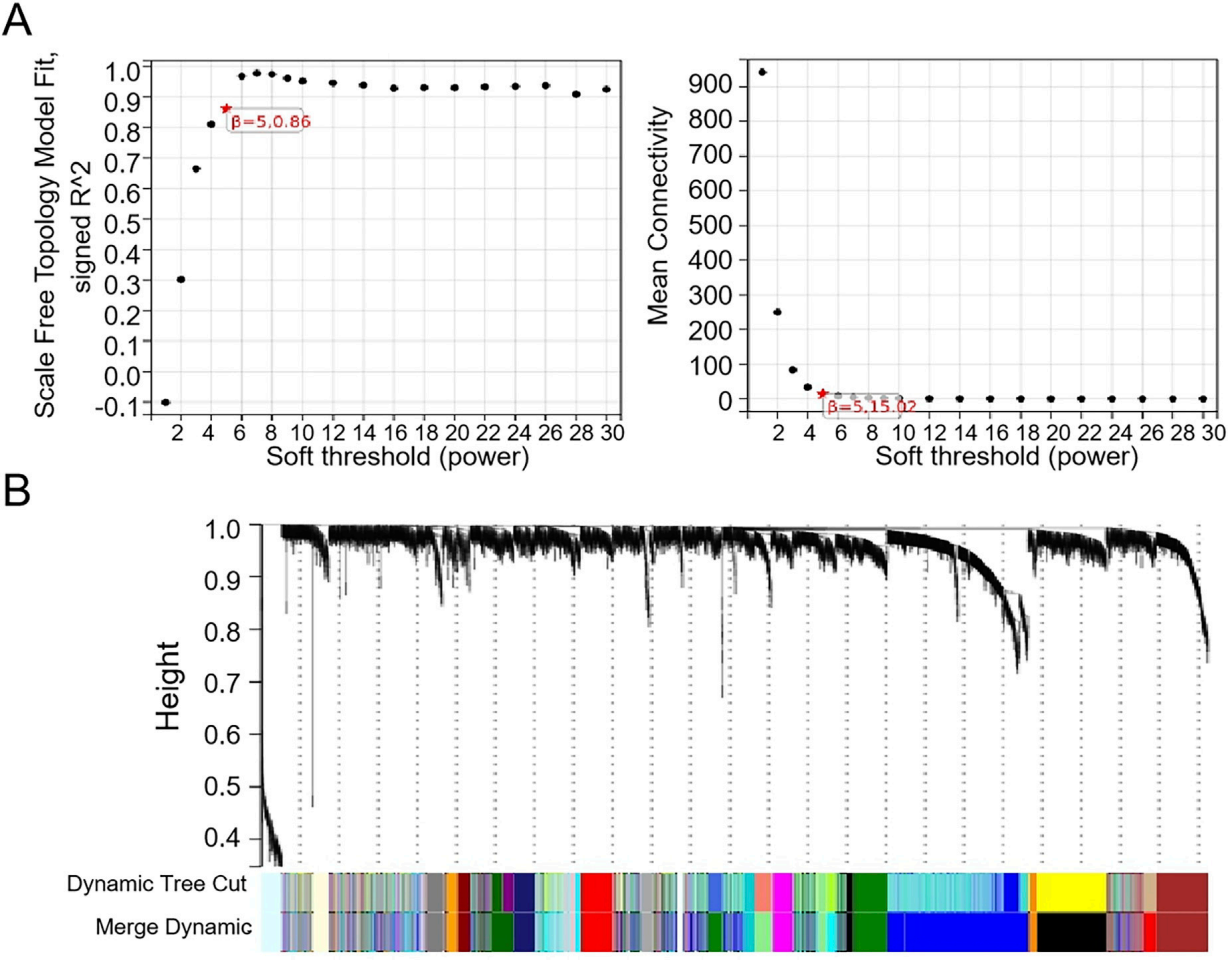
WGCNA of the merged dataset. **(A)** β = 5 is chosen as the soft threshold based on the scale independence and average connectivity. **(B)** Dendrogram of all genes clustered based on a dissimilarity measure (1-TOM).

**FIGURE 3:**
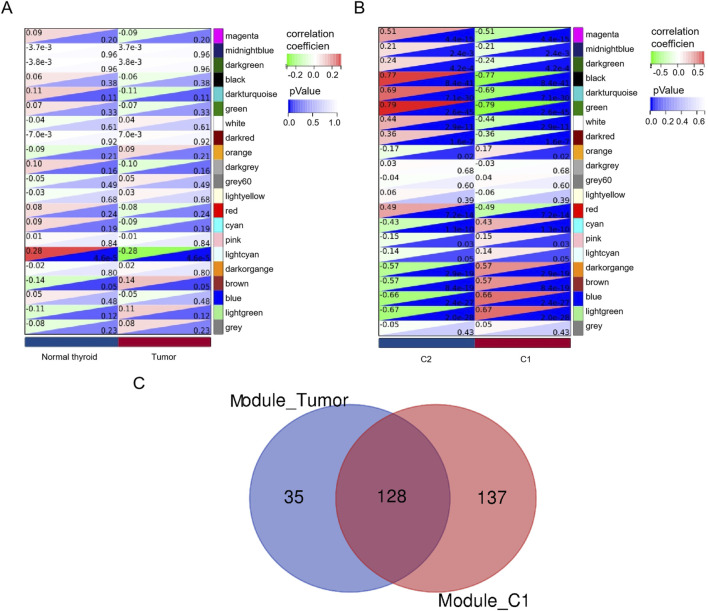
Identification of hub genes. A heatmap of the correlation between module genes and tumor module **(A)** or/C1 and **(B)** C2. For each pair, the top left triangle is colored to represent the correlation coefficient; the bottom right one is colored to indicate the P-value. **(C)** Venn analysis of overlapping genes.

The WGCNA was used to analyze the correlation between gene modules and groups/clusters. In this study, we found that when the soft threshold β was set to 5, the constructed gene co-expression network had approximately a scale-free topological distribution, and the fitting degree *R* ^2^ = 0.86 (Figure 2A). Therefore, a soft threshold β = 5 was employed to establish the scale-free network, resulting in the identification of 21 modules (Figure 2B). Among these, 163 genes exhibited a strong correlation with the tumor groups and were selected as hub genes (MM > 0.8, P < 0.05). Additionally, 265 genes displayed a strong correlation with cluster 1 (C1) and were identified as hub genes (MM > 0.8, P < 0.05), while another set of 34 genes showed a strong correlation with cluster 2 (C2) and were designated as the hub module (MM > 0.8, P < 0.05). Based on the results of the four-fold table diagnostic test (Table 2), we find that 109 samples in C1 are classified as the tumor group, and 17 samples are classified as the normal group. Sixty-two samples from C2 were classified as the tumor group, and 28 samples were classified as the normal group. The sensitivity of the consistency clustering analysis diagnostic test was 64%, and the specificity was 62%. The area under the ROC curve (AUC) was 0.63, with a positive likelihood ratio of 1.7 and a negative likelihood ratio of 0.583. The prevalence of the population being tested stood at 79.2%, yielding a positive predictive value (PPV) of 86.5% and a negative predictive value (NPV) of 31.1%. The overall accuracy reached 63.4%. This suggests that the consistency clustering analysis model holds a favorable diagnostic value. To sum up, the characteristics of C1 are more aligned with reality. As a result, Venn analysis identified 128 genes as common genes between the tumor module and the C1 module (Figure 3C).

**TABLE 2:** Four-fold table diagnostic test for gene expression to predict risk for patients with THCA.

Group		Merged dataset		Total
		Tumor	Normal	
Predicted	C1	109	17	126
	C2	62	28	90
Total		171	45	216

C1, cluster 1; C2, cluster 2.

### 3.3 GO and KEGG analysis of 128 genes

**FIGURE 4:**
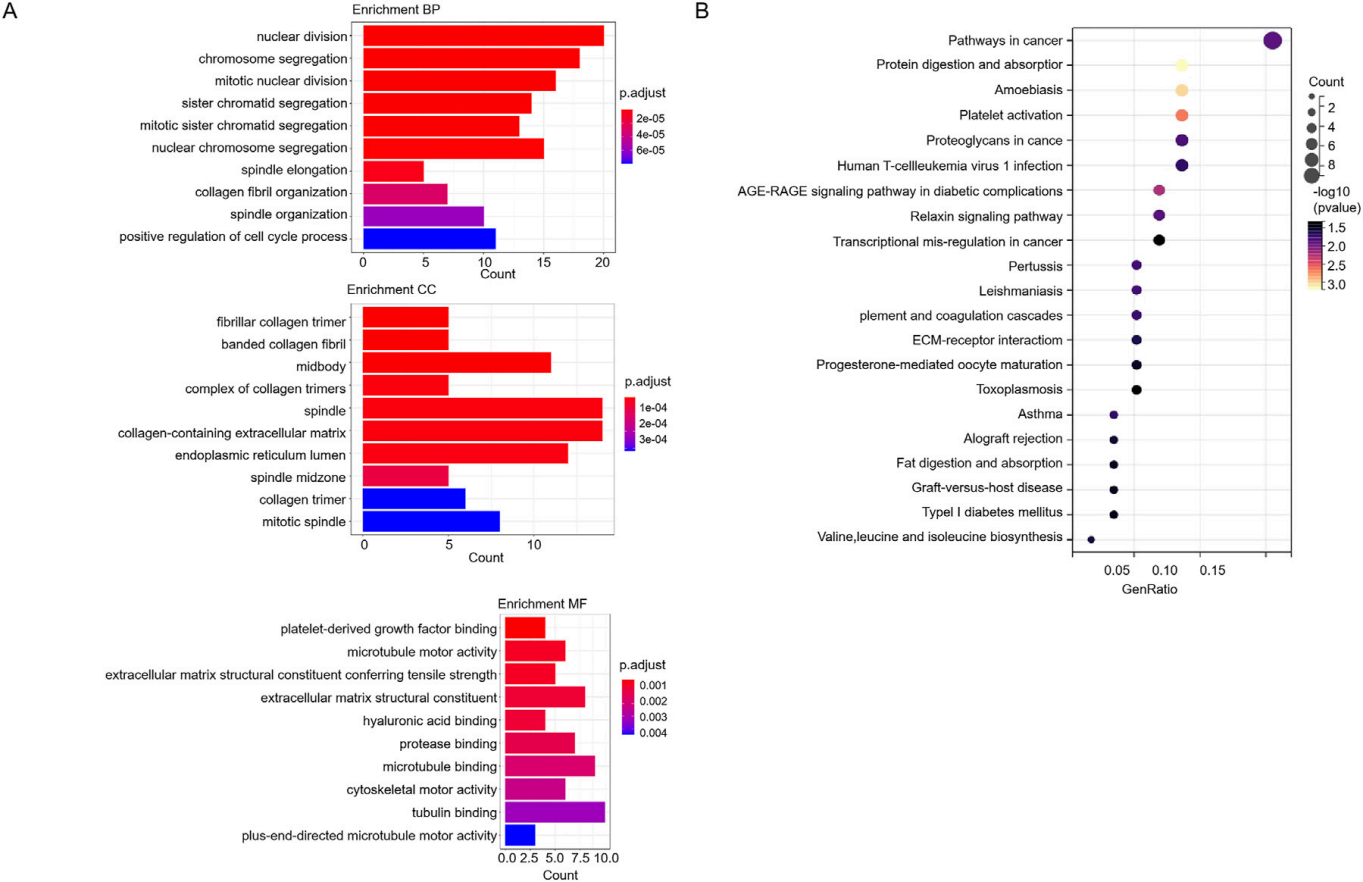
GO and KEGG analysis of 128 genes. **(A)** The GO analysis of 128 genes. **(B)** The KEGG pathway analysis of 128 genes.

GO analysis showed that 128 genes were enriched in nuclear division, positive regulation of cell cycle process, collagen-containing extracellular matrix, mitotic spindle, platelet-derived growth factor binding, microtubule motor activity, and cytoskeletal motor activity (Figure 4A). The 128 genes were significantly related to pathways in cancer and transcriptional misregulation in cancer (Figure 4B).

### 3.4 Identification of lymph node metastasis markers in PTC

**FIGURE 5:**
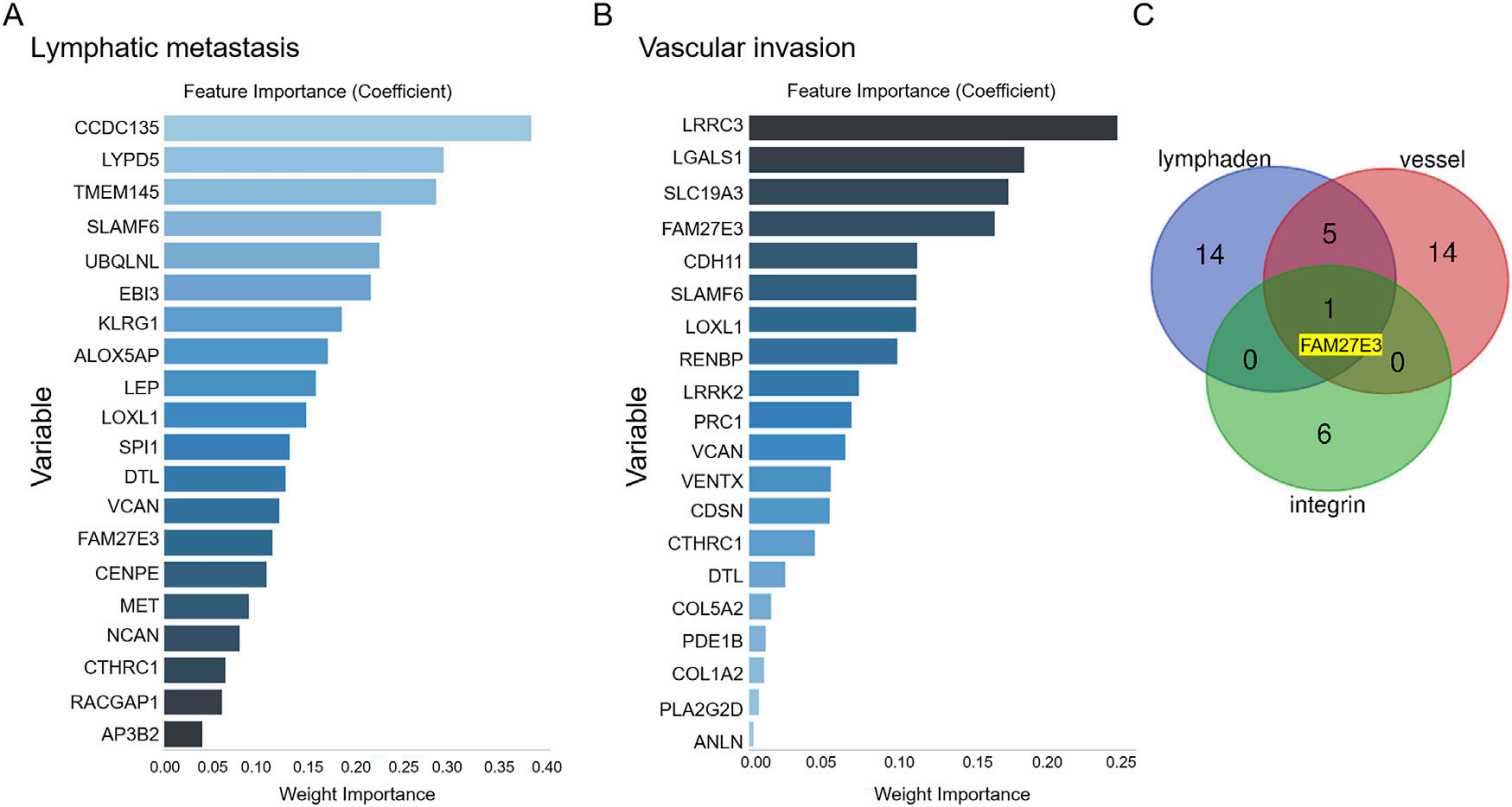
Identification of lymph node metastasis-related hub genes in PTC. After obtaining data for 128 gene expressions from the testing set, the LASSO algorithm identifies the top 20 influential factors associated with PTC lymph node metastasis **(A)** or vascular invasion **(B)**. **(C)** Venn analysis of hub genes related to PTC lymph node metastasis.

First, we obtained 128 gene expressions from the PTC-related dataset (GSE60542: 33 PTC patients and 23 PTC lymph node metastasis patients; Table 3). This dataset encompasses two distinct outcome events: metastasis and vascular invasion. The LASSO algorithm was employed to assess the impact of the 128 genes on these two events, resulting in the selection of the top 20 genes (Figures 5A,B). Subsequently, a Venn analysis was conducted with the seven integrin genes and the above genes to identify overlapping genes. FAM27E3 is the only overlapping gene (Figure 5C).

**TABLE 3:** The information of GSE60542 dataset.

Variables	Category	PTC (n = 33)	PTC lymph node metastasis (n = 23)
Gender	Female	19	13
	Male	14	10
Age	<55	26	17
	≥55	7	6
Stage	I	19	17
	III	10	4
	IV	3	2
Vascular_invasion	No	28	17
	Yes	3	4

### 3.5 FAM27E3 is a lymph node metastasis marker in PTC

**FIGURE 6:**
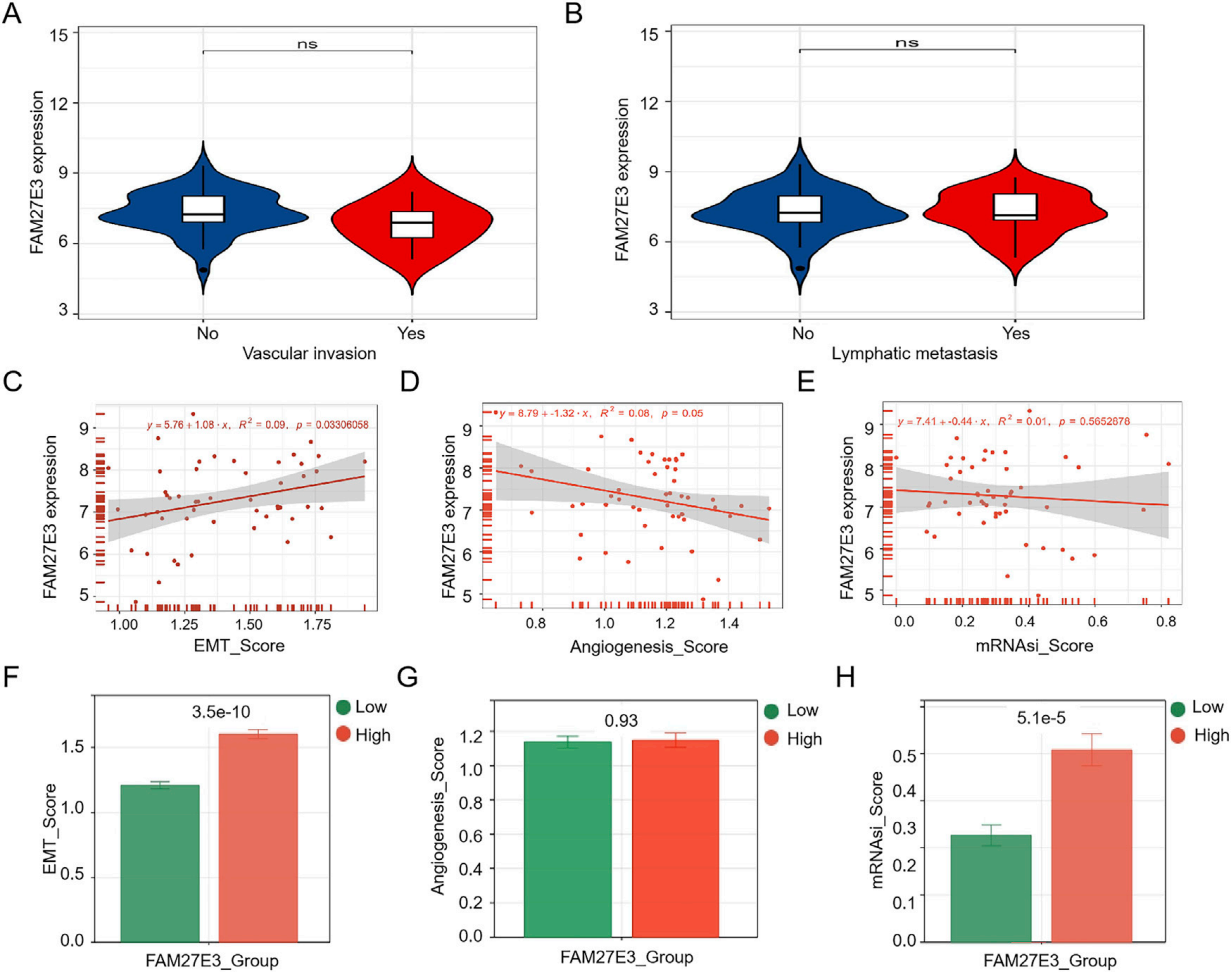
Characteristic analysis of FAM27E3. The expression of FAM27E3 in vascular invasion or no **(A)** and in lymphatic metastasis or no **(B)**. The correlation analysis between FAM27E3 expression and EMT_score **(C)**, angiogenesis_score **(D)**, and mRNAsi_score **(E)**. The differential analysis of EMT_score **(F)**, angiogenesis_score **(G)**, and mRNAsi_score **(H)** between the FAM27E3 high-expression and low-expression groups. P < 0.05 is considered statistically significant.

Score analysis was used to identify the relationship between the FAM27E3 expression and lymph node metastasis. The expression level of FAM27E3 showed no significant difference between the metastatic and non-metastatic groups, as well as between the invasive and non-invasive groups (Figures 6A,B). The expression of FAM27E3 exhibits a significant positive correlation with the EMT score but lacks a substantial correlation with the angiogenesis and mRNAsi scores (Figures 6C–E). The FAM27E3 high-expression group had higher EMT score (Figure 6F) and mRNAsi scores (Figure 6H). No disparities in angiogenesis score between the high- and low-expression groups of FAM27E3 were found (Figure 6G).

### 3.6 FAM27E3 affects lymph node metastasis of PTC via regulating macrophage infiltration

**FIGURE 7:**
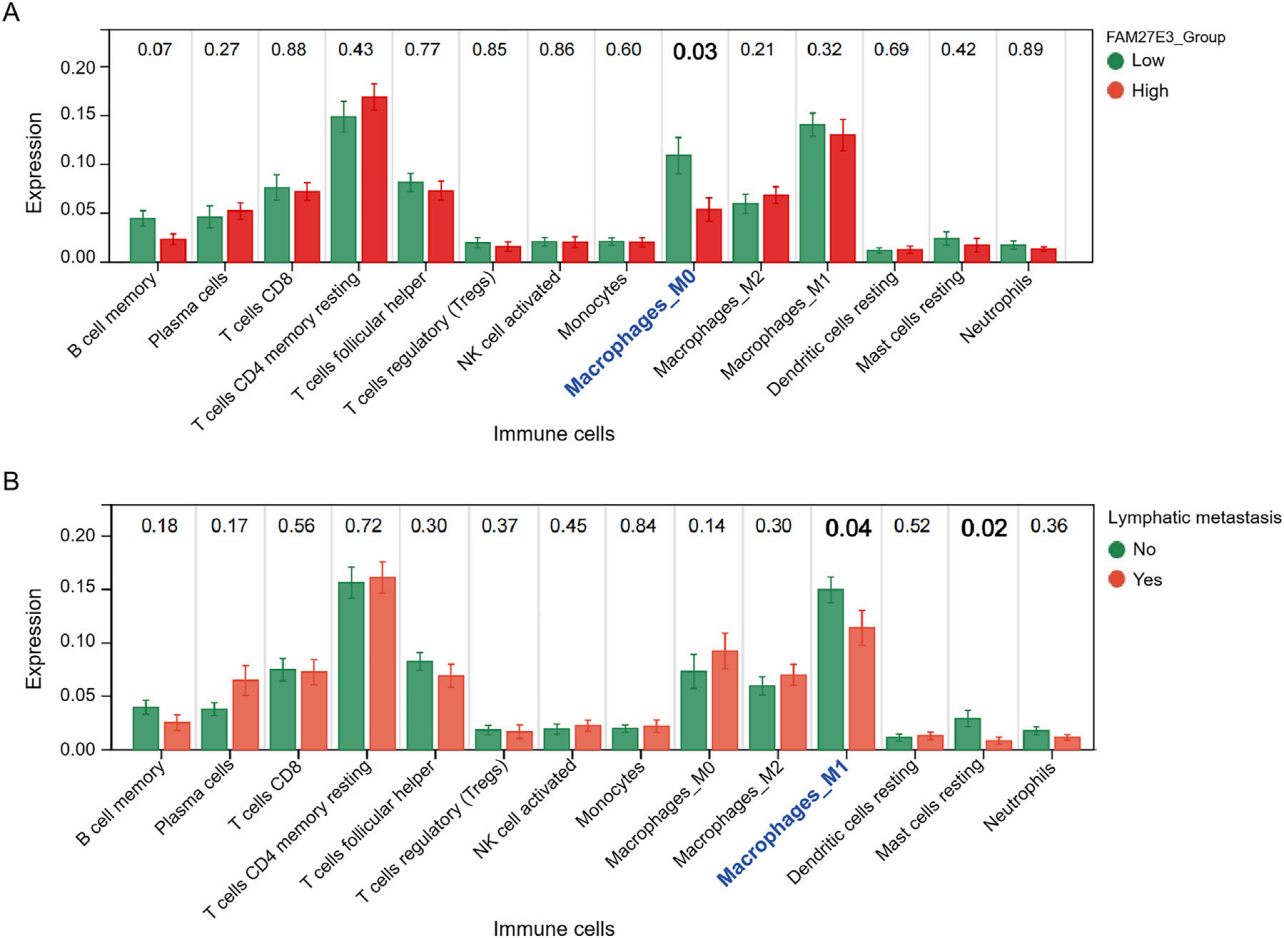
Immune cell infiltration analysis of FAM27E3. **(A)** Immune cell infiltration analysis between the FAM27E3 high-expression and low-expression groups. **(B)** Immune cell infiltration analysis between the lymphatic metastasis group and the non-metastasis group.

Immune cell infiltration analysis revealed a significantly lower level of macrophages in the high-expression group of FAM27E3 than in the low-expression group (Figure 7A). Similarly, macrophage levels in the group without lymph node metastasis were lower than those in the group with lymph node metastasis (Figure 7B), and the level of M1 macrophages was notably reduced in the group with lymph node metastasis (Figure 7B). These findings suggested that FAM27E3 may contribute to lymph node metastasis in PTC by modulating the infiltration of M1 macrophages. To explore the clinical value of FAM27E3 in predicting lymph node metastasis, we performed ROC analysis based on the TCGA-THCA dataset. ROC results showed that FAM27E3 had a certain clinical value in predicting lymphatic metastasis (AUC: 0.66, 95% CI: 0.61–0.71, sensitivity: 0.46, specificity: 0.82, Supplementary Figure S1).

### 3.7 FAM27E3 knockdown attenuates carcinogenic phenotype of thyroid cancer cells

**FIGURE 8:**
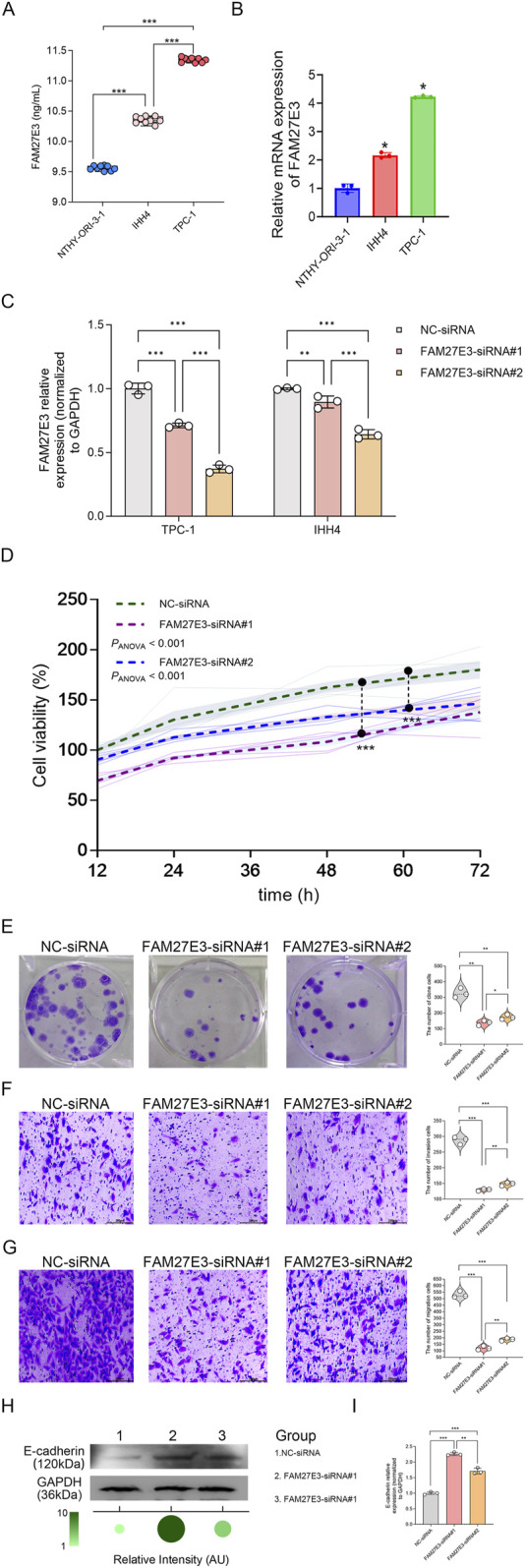
The effect of FAM27E3 on the proliferation, migration, and invasion in PTC cells. **(A)** ELISA analysis of FAM27E3 expression in protein levels. **(B)** RT-PCR analysis of FAM27E3 expression in control and PTC cell lines. **(C)** RT-PCR analysis of FAM27E3 expression in FAM27E3-siRNA#1- or #2-transfected PTC cells. In FAM27E3-siRNA#1 transfected PTC cells **(D),** CCK8 assay of cell viability **(E),** clone formation assay of cell proliferation, Transwell analysis of the invasion **(F)** and migration **(G)**, Western blot **(H),** and RT-PCR **(I)** analysis of E-cadherin. The experiment was repeated three times. *P < 0.05 is considered statistically significant.

ELISA analysis revealed elevated levels of FAM27E3 protein expression in PTC cell lines TPC-1 and IHH4 compared to normal NTHY-ori-3.1 cells (normal NTHY-ori-3.1 cells vs. TPC-1 vs. IHH4: 9.5 vs. 11.5 vs. 10.0 ng/mL, Figure 8A). RT-PCR analysis showed that FAM27E3 mRNA expression in PTC cell lines TPC-1 and IHH4 was elevated compared to normal NTHY-ori-3.1 cells (Figure 8B). These results indicated the high expression of FAM27E3 in PTC.

We then explored the effect of FAM27E3 expression inhibition on the behaviors of cancer cells. Transient knockdown of FAM27E3 in TPC-1 cells was performed with siRNAs #1 and #2, finding that it reduced FAM27E3 expression by approximately 60% and 40%, respectively. However, FAM27E3 knockdown in IHH4 cells using siRNAs #1 and #2 showed no better effect than TPC-1 cells (Figure 8C). Consequently, we selected TPC-1 as the primary cancer cell line for subsequent experiments. Functional assessment demonstrated that FAM27E3 silencing led to a significant decrease in cell viability to 50% of control levels at 72 h post-transfection and reduced clone formation ability by approximately 60% compared to non-targeted controls (Figure 8D). The colony formation results showed that the clone formation ability of the cells was inhibited in the FAM27E3-siRNAs #1 and FAM27E3-siRNAs #2 (Figure 8E). Transwell assays indicated a three-fold decrease in the invasiveness and migration of silenced cells (Figures 8F,G). Western blot analysis confirmed a notable increase in E-cadherin levels in the siRNA-FAM27E3-treated groups (Figure 8H). RT-PCR results revealed upregulation of E-cadherin mRNA in the knockdown group (Figure 8I). Furthermore, both RT-PCR and Western blot analyses demonstrated that FAM27E3-siRNA #1 significantly enhanced E-cadherin expression compared to FAM27E3-siRNA #2 (Figures 8H,I). Collectively, these results suggested that silencing of FAM27E3 disrupts the proliferation, clone formation, and metastatic capabilities of thyroid cancer cells, highlighting its crucial role in maintaining oncogenic phenotypes.

### 3.8 FAM27E3 is involved in the p53 signaling pathway in lymph node metastasis of PTC

**FIGURE 9:**
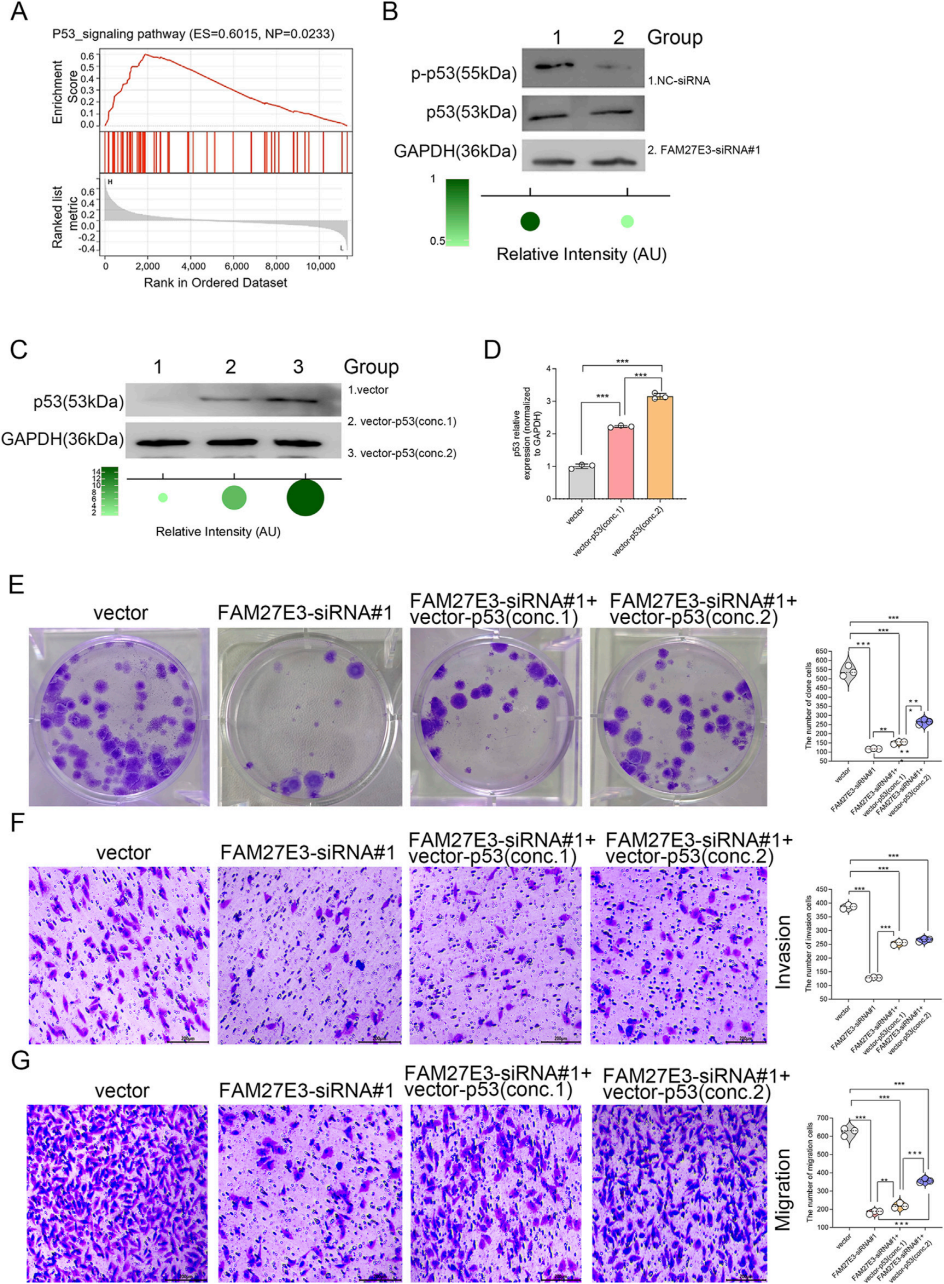
FAM27E3 is involved in the p53 signaling pathway in lymph node metastasis of PTC. **(A)** GSEA analysis of FAM27E3. **(B)** Western blot analysis of the phosphorylation level of p53. Western blot **(C)** and RT-PCR **(D)** analysis of p53 expression after a vector expressing p53 (two concentrations) was transfected into TPC-1 cells. **(E)** Clone formation assay of cell proliferation, Transwell analysis of the invasion **(F)** and migration **(G)**. The experiment was repeated three times. *P < 0.05 is considered statistically significant.

Gene set enrichment analysis (GSEA) identified a significant enrichment of the p53 signaling pathway associated with FAM27E3 (normalized P-value = 0.0233), implying a regulatory link between FAM27E3 and p53 in THCA (Figure 9A). Western blot analysis demonstrated a notable decrease in p53 protein phosphorylation levels in TPC-1 cells following FAM27E3 silencing using siRNA#1 compared to the control group (Figure 9B). Overexpression of p53 in TPC-1 cells through transfection with increasing concentrations of Vector-p53 resulted in a dose-dependent upregulation of p53 expression, with the most pronounced elevation observed in the group treated with the highest concentration (conc.2) (Figures 9C,D). Functional assays revealed that FAM27E3 knockdown (siRNA#1) led to an approximately 60% decrease in colony formation, which could be partially rescued by high-concentration Vector-p53 (conc.2) treatment, as depicted in Figure 9E. Subsequently, Transwell experiments validated that FAM27E3 silencing (siRNA#1) reduced the number of invasive cells and migration capacity by approximately three-fold, and high-concentration Vector-p53 (conc.2) restored approximately 50% of the migration and invasion capabilities (Figures 9F,G). Collectively, these findings suggested that FAM27E3 promoted tumor progression by counteracting the p53 pathway, and its suppression can activate the p53-mediated tumor-suppressive effects, thereby impeding the proliferation and metastasis of thyroid cancer.

### 3.9 FAM27E3 interferes with TP53 activity, which promotes the malignant phenotype deterioration of the tumor

**FIGURE 10:**
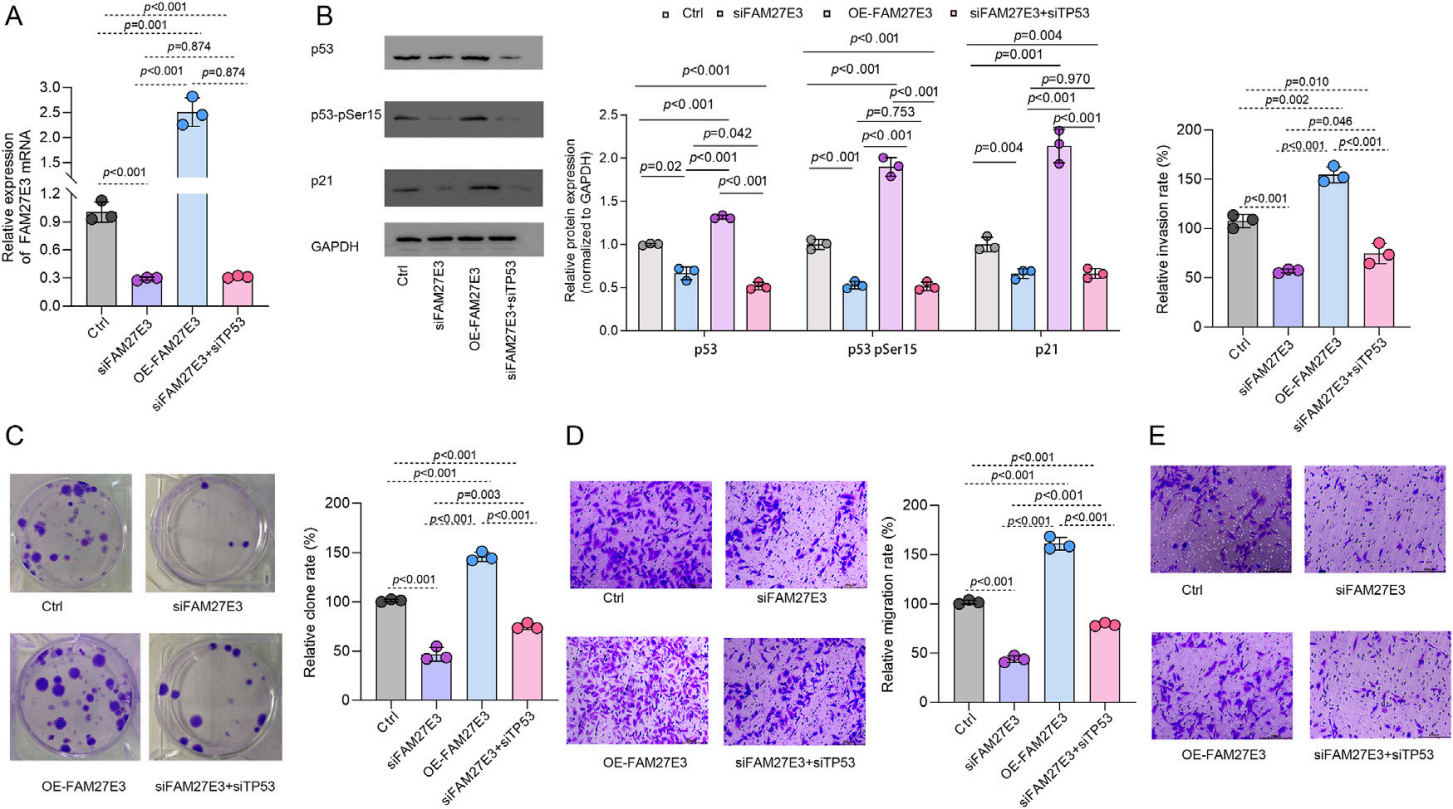
FAM27E3 interferes with TP53 activity and thus affects tumor development. **(A)** RT-PCR and **(B)** Western blot analysis of Ctrl, siFAM27E3, OE-FAM27E3, and siFAM27E3+siTP53. **(C)** Clone formation assay of cell proliferation. Transwell analysis of the **(D)** migration and **(E)** invasion. The experiment was repeated three times. *P < 0.05 is considered statistically significant.

Functional experiments based on the P53 wild-type TPC-1 cell model showed that the expression level of FAM27E3 significantly regulated the p53 signaling pathway activity and the cell malignant phenotype. RT-PCR and Western blot analysis showed that specific silencing of FAM27E3 (siFAM27E3 group) resulted in a significant decrease in the level of Ser15, a key phosphorylation site of p53, and a significant decrease in the expression of its downstream effector molecule p21. In contrast, FAM27E3 overexpression (OE-FAM27E3 group) increased p53-Ser15 phosphate level and p21 expression. These results suggested that FAM27E3 expression may interfere with the activity of TP53 (Figures 10A,B). In the phenotypic experiment, compared with the control group, silencing FAM27E3 reduced the clone formation rate and Transwell invasion/metastasis ability, while overexpressing FAM27E3 significantly increased the clone formation and invasion/metastasis ability (p < 0.05). Co-silencing of FAM27E3 and TP53 (siFAM27E3 + siTP53 group) could partially reverse the phenotypic inhibition effect: clone formation and invasion and metastasis ability were restored to about 80% of that of the control group, indicating that FAM27E3’s promotion of cancer is partly dependent on p53 functional inactivation (Figures 10C–E).

## 4 Discussion

Our previous research has shown that the upregulation of integrin-related gene FAM27E3 is associated with an unfavorable prognosis (Zhang et al., 2023). In this study, our findings validated the high expression of FAM27E3 in PTC cell lines and its pivotal role in PTC lymph node metastasis. FAM27E3, also known as FAM-interleukin-27 receptor subunit 3, is a potential drug target or biomarker associated with various diseases, including cancer, neurological disorders, and immune system disorders (Zhang et al., 2023; Gong et al., 2024; Zhou et al., 2022). Functionally, it interacts with cytokine receptors such as interleukin-1 and tumor necrosis factor to modulate cellular immunity and signal transmission between immune cells and neurons. While there was no statistically significant difference in FAM27E3 expression between the PTC lymph node metastatic and non-metastatic groups, this may be attributed to the limited sample size. However, further analysis revealed a notably higher EMT score (Dai et al., 2023) in the high-expression group of FAM27E3 than in the low-expression group, with FAM27E3 expression levels showing a significant positive correlation with the EMT score. Additionally, the mRNAsi score (Zhang et al., 2020) was markedly elevated in the high-expression group of FAM27E3 compared to the low-expression group. The EMT is a critical process in cancer cell metastasis, leading to enhanced cell mobility and migration ability as epithelial cells acquire mesenchymal characteristics (Pastushenko et al., 2018). FAM27E3 plays a crucial role in physiological processes such as embryonic development, wound healing, and tissue regeneration, while also being involved in pathological processes like tissue fibrosis and the progression of malignant tumors (Zhang et al., 2023; Gong et al., 2024; Zhou et al., 2022). Additionally, FAM27E3 promotes EMT, facilitating tumor cell invasion and migration to distant tissues, as well as conferring stem-like properties upon migrated tumor cells that contribute to the formation of macroscopic metastatic lesions and the development of multidrug resistance. These collective findings indicate that FAM27E3 may enhance tumor stemness through EMT to promote lymph node metastasis. Our experimental results further demonstrated significant upregulation of FAM27E3 in PTC cell lines and substantial inhibition of PTC cell proliferation, migration, and invasion upon its silencing. Consequently, these research outcomes suggest that FAM27E3 could potentially serve as a marker for lymph node metastasis in PTC.

Increasing research evidence supports the interaction between cancer stem cells and immune cells within the tumor microenvironment, encompassing the impact of cancer stem cells on tumor-associated macrophages, myeloid-derived suppressor cells, and T cells (Muller et al., 2020). Additionally, it underscores the importance of these immune cells in preserving the stemness of cancer stem cells and their ecological niche for survival. Likewise, it can be inferred that cancer cells exhibiting stem-like characteristics are closely associated with immune cells (Desai et al., 2023; Wu et al., 2023). The analysis of immune cell infiltration related to FAM27E3 indicates a lower level of macrophages in the high-expression group of FAM27E3. There is also a decrease in M1 macrophage levels in the PTC lymph node metastasis group. Macrophages participate in tumor lymphangiogenesis and play a pivotal role in lymphatic metastasis. They consist of at least two subpopulations, M1 and M2. While M1 macrophages have an anti-tumor function, M2 macrophages possess various pro-tumor functions, such as immune suppression, angiogenesis, and neovascularization (Yunna et al., 2020). Although the level of M2 macrophages did not show statistical significance between the FAM27E3 high-expression group and the lymph node metastasis group, it is evident that the level of M2 macrophages was higher in both the FAM27E3 high-expression group and the lymph node metastasis group. In summary, the high expression of FAM27E3 promotes polarization of macrophages toward the M2 phenotype and facilitates PTC lymph node metastasis.

We also investigated the mechanism of FAM27E3 impacting lymph node metastasis in PTC. GSEA revealed that FAM27E3 was related to the p53 signaling pathway, and we further validated that its silencing reduced the p-p53 protein expression. These results contradict classical tumor suppression theory, wherein wild-type p53 typically suppresses EMT through E-cadherin induction and inhibits M2 macrophage polarization via the p53/MDM2/c-MYC axis (Li et al., 2015). Paradoxically, FAM27E3 silencing (with concomitant p53 downregulation) suppressed tumor migration and invasion, whereas FAM27E3 overexpression (with elevated p53) increased both EMT scores and M2 macrophage enrichment. Notably, PTC specimens exhibit progressively higher p53 positivity with advancing pathology (Abdelhafez et al., 2023), consistent with reports that mutant p53 acquires oncogenic functions: direct activation of EMT transcription factors (Adorno et al., 2009), TGF-β-mediated induction of M2 polarization (Cooks et al., 2013), and immune microenvironment reprogramming through exosomal miRNAs such as miR-1246 (Weissmueller et al., 2014). We therefore hypothesize that elevated p53 in our system reflects mutant p53 activity, with FAM27E3 potentially stabilizing mutant p53 or enhancing its transcriptional output to drive EMT and M2 polarization, ultimately facilitating lymph node metastasis. In this study, we confirmed that FAM27E3 can influence the TP53 activity, and inhibition of TP53 can reverse the inhibitory effect of FAM27E3 downregulation on the malignant phenotype of the tumor. These findings suggested that FAM27E3 may affect the malignant phenotype of tumors by regulating the activity of TP53, and TP53 may be a key downstream target for FAM27E3 in regulating the malignant phenotype of tumors. The specific molecular mechanism by which FAM27E3 regulates TP53 (such as direct action or indirect regulation) needs to be further clarified in future research, as does the universality of this regulation in different tumor types.

This study provides the first comprehensive functional characterization of FAM27E3 in PTC metastasis, integrating cellular assays, EMT/mRNAsi scoring, and immune profiling to propose the novel FAM27E3-p53-EMT axis. However, there are still several limitations. First, the functional status of the key molecule p53 is not clear, and the lack of mutation detection data makes it difficult to distinguish its pro-oncogenic/tumor suppressor role. Second, the specific mechanism of FAM27E3 regulating p53 and its effector molecules has not been resolved; in addition, the study relies on cell line models and lacks animal *in vivo* lymph node metastasis experimental verification, while the immune interaction mechanism is only speculated through bioinformatics analysis and should be supplemented with tumor cell–macrophage co-culture experiments. At the clinical level, the small sample size of the lymph node metastasis group may affect the statistical power, and the retrospective single-center design requires a prospective multicenter cohort to verify the value of the marker. Therefore, future work will focus on using gene editing technology to construct a p53 mutation model to clarify the necessity, develop targeted inhibitors to evaluate the therapeutic potential, and use single-cell sequencing to analyze the spatiotemporal interaction network between tumor cells and the immune microenvironment.

## 5 Conclusion

FAM27E3 expression is upregulated in PTC cell lines. FAM27E3 silencing inhibits PTC cell proliferation, migration, and invasion and suppresses phosphorylated p53 expression. There is a significant positive correlation between FAM27E3 expression and the EMT score, with higher EMT and mRNAsi scores observed in the high-expression group of FAM27E3. Additionally, FAM27E3 plays a role in promoting macrophage polarization toward the M1 phenotype. Consequently, FAM27E3 serves as a marker for PTC lymph node metastasis. The mechanism may involve regulating p53 phosphorylation and affecting its signaling pathway. However, given the contradiction between the tumor suppressor properties of the p53 pathway and the cancer-promoting phenotype of FAM27E3, it is necessary to further clarify the p53 mutation status and the specific mechanism of FAM27E3.

## Data Availability

The raw data supporting the conclusions of this article will be made available by the authors, without undue reservation.

## Abbreviations

ANOVA, one-way analysis of variance; AUC, area under curve; CDF, cumulative distribution function; DTC, differentiated thyroid cancer, MTC, medullary thyroid cancer; ECM, extracellular matrix; EMT, epithelial–mesenchymal transition; ESCs, embryonic stem cells; FBS, fetal bovine serum; FTC, follicular thyroid carcinoma; GSEA, gene set enrichment analysis; HTC, Hurthle cell carcinoma; ILK, integrin-linked kinase; LASSO, least absolute shrinkage and selection operator; NPV, negative predictive value; PCA, principal component analysis; PCMC, progenitor cell biology consortium; PPV, positive predictive value; PSCs, pluripotent stem cells; PTC, papillary thyroid carcinoma; ROC, receiver operating characteristic; THCA, thyroid cancer; WGCNA, weighted gene co-expression network analysis; WHO, World Health Organization
