# Supplementary figures and images for: Integrin factor (FAM27E3), as a metastatic marker of papillary thyroid carcinoma, through the p53 signaling pathway promoting lymph node metastasis
Source: Front Genet. 2025 Jul 30;16:1593553. doi: 10.3389/fgene.2025.1593553 (PMC12343231; doi:10.3389/fgene.2025.1593553)

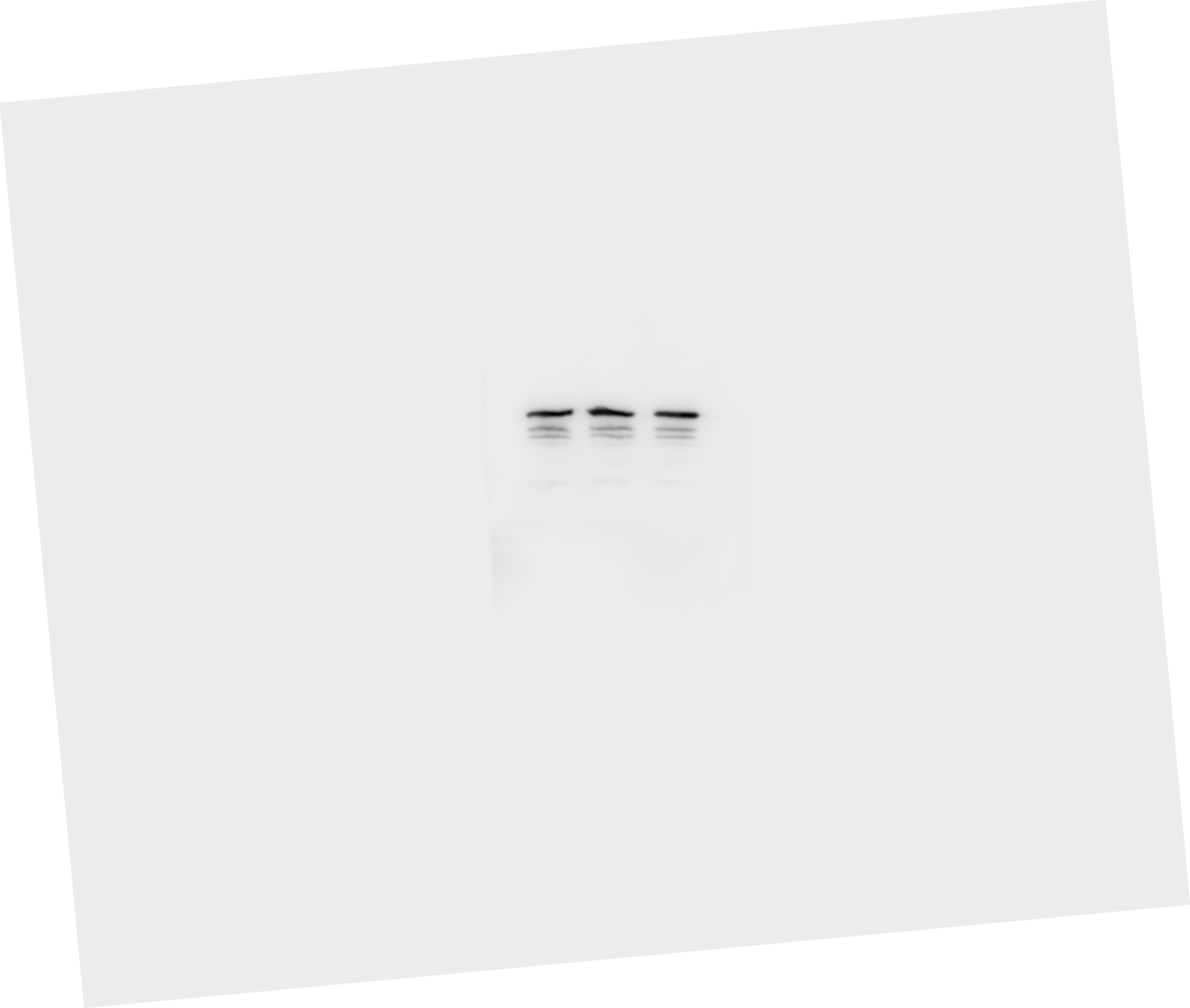

Supplement: Supplementary file 1 [file DataSheet1.zip › WB data/GAPDH(1).tif]

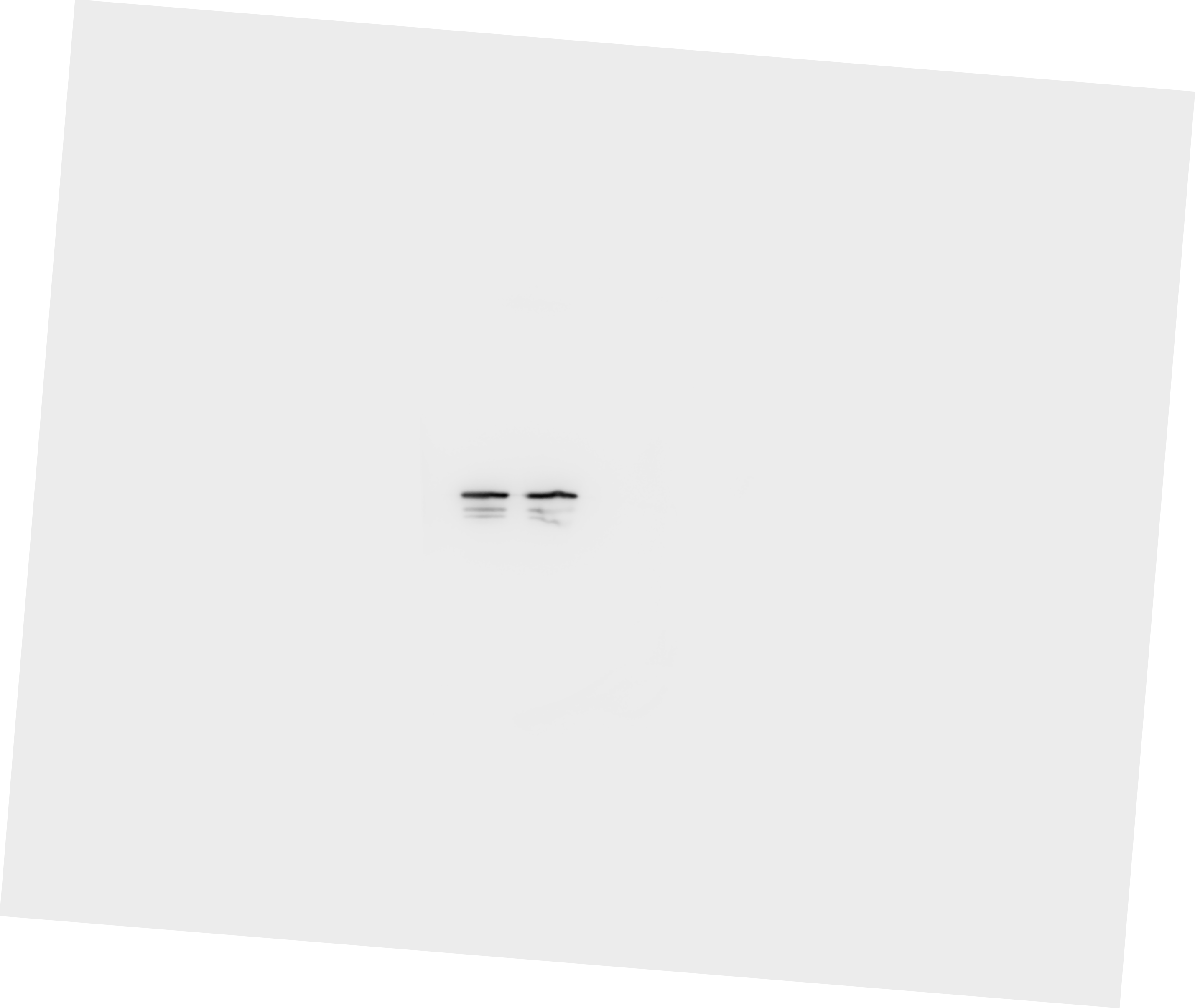

Supplement: Supplementary file 1 [file DataSheet1.zip › WB data/GAPDH(2).tif]

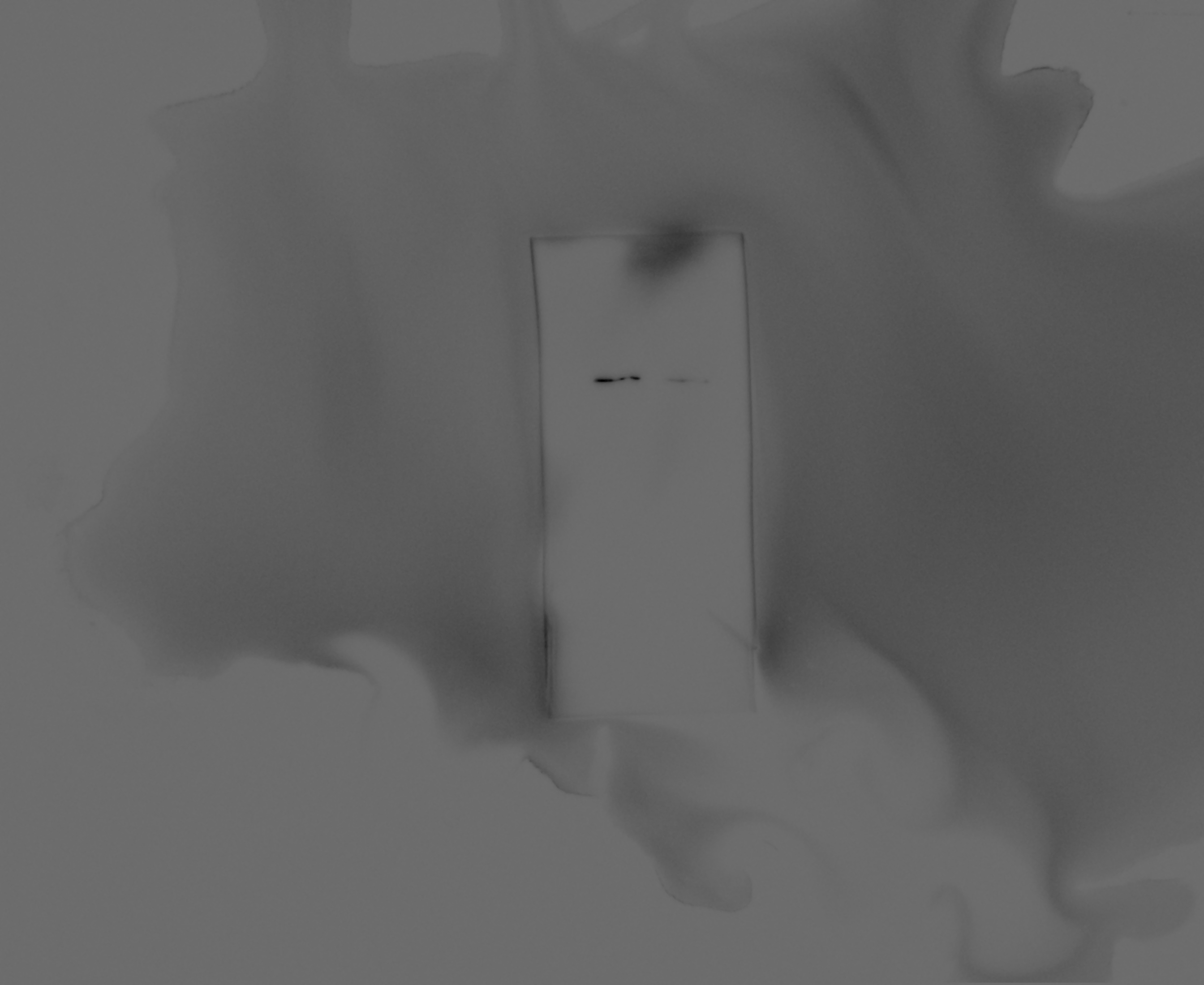

Supplement: Supplementary file 1 [file DataSheet1.zip › WB data/p-P53.tif]

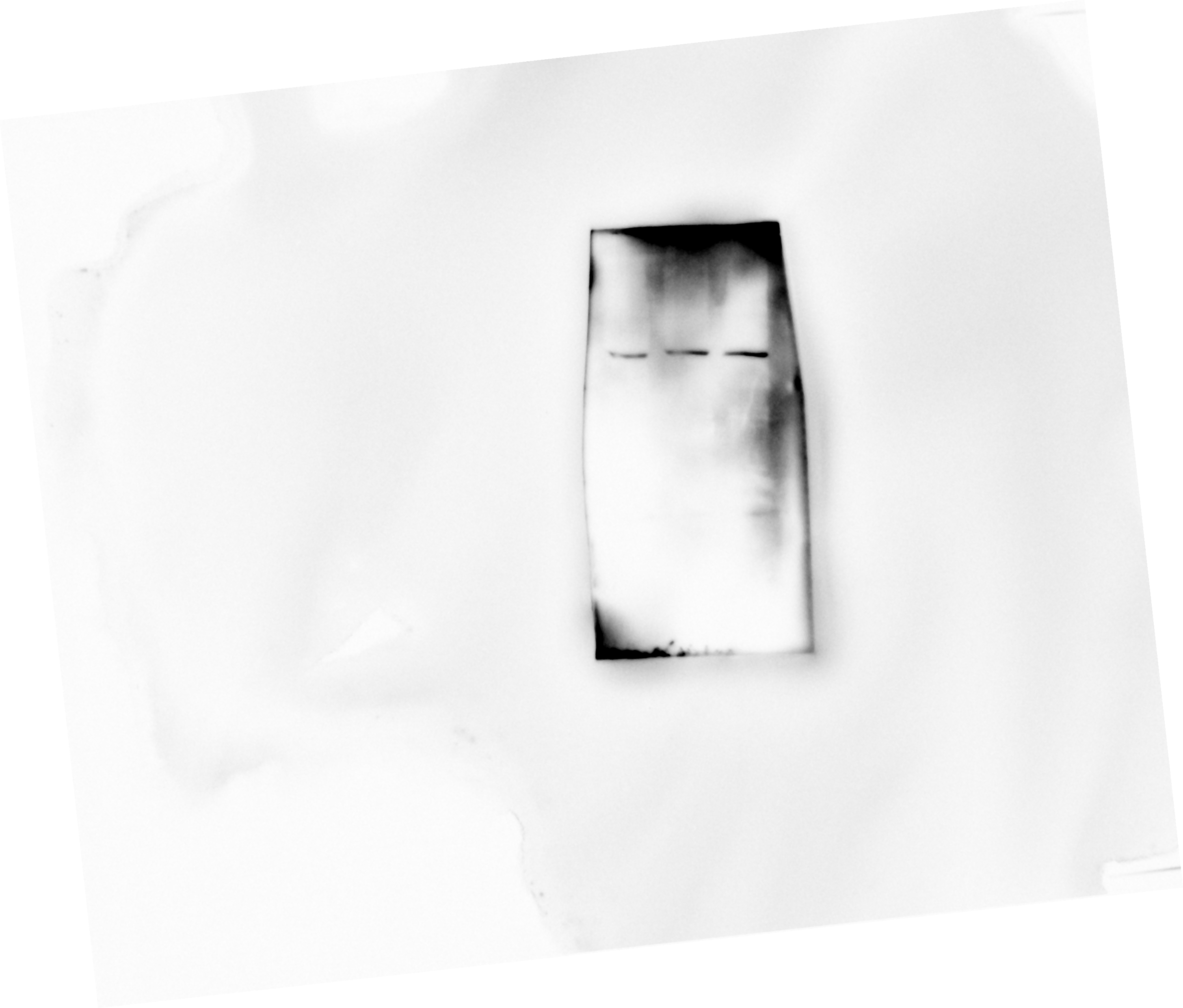

Supplement: Supplementary file 1 [file DataSheet1.zip › WB data/P53(1).tif]

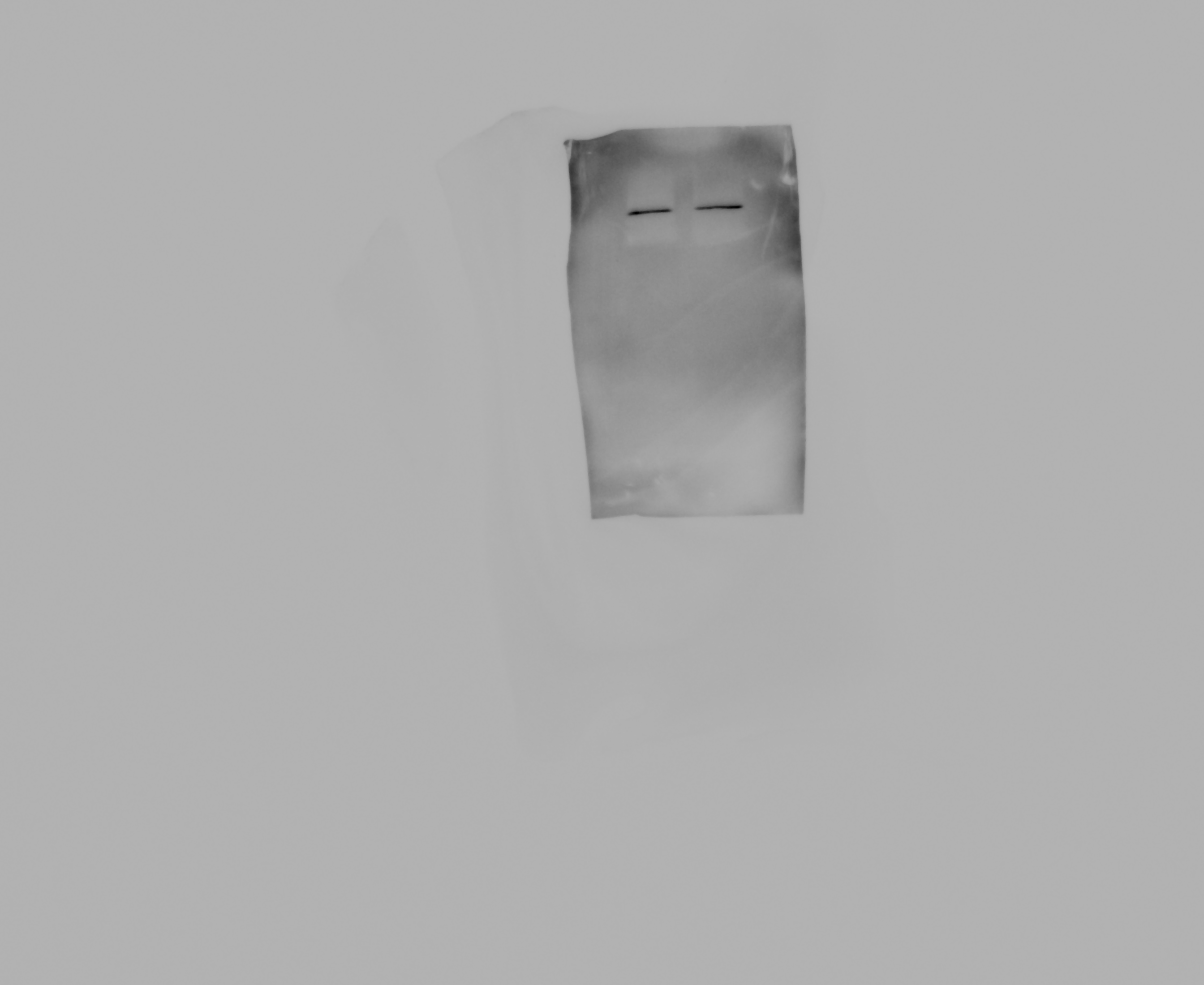

Supplement: Supplementary file 1 [file DataSheet1.zip › WB data/P53(2).tif]

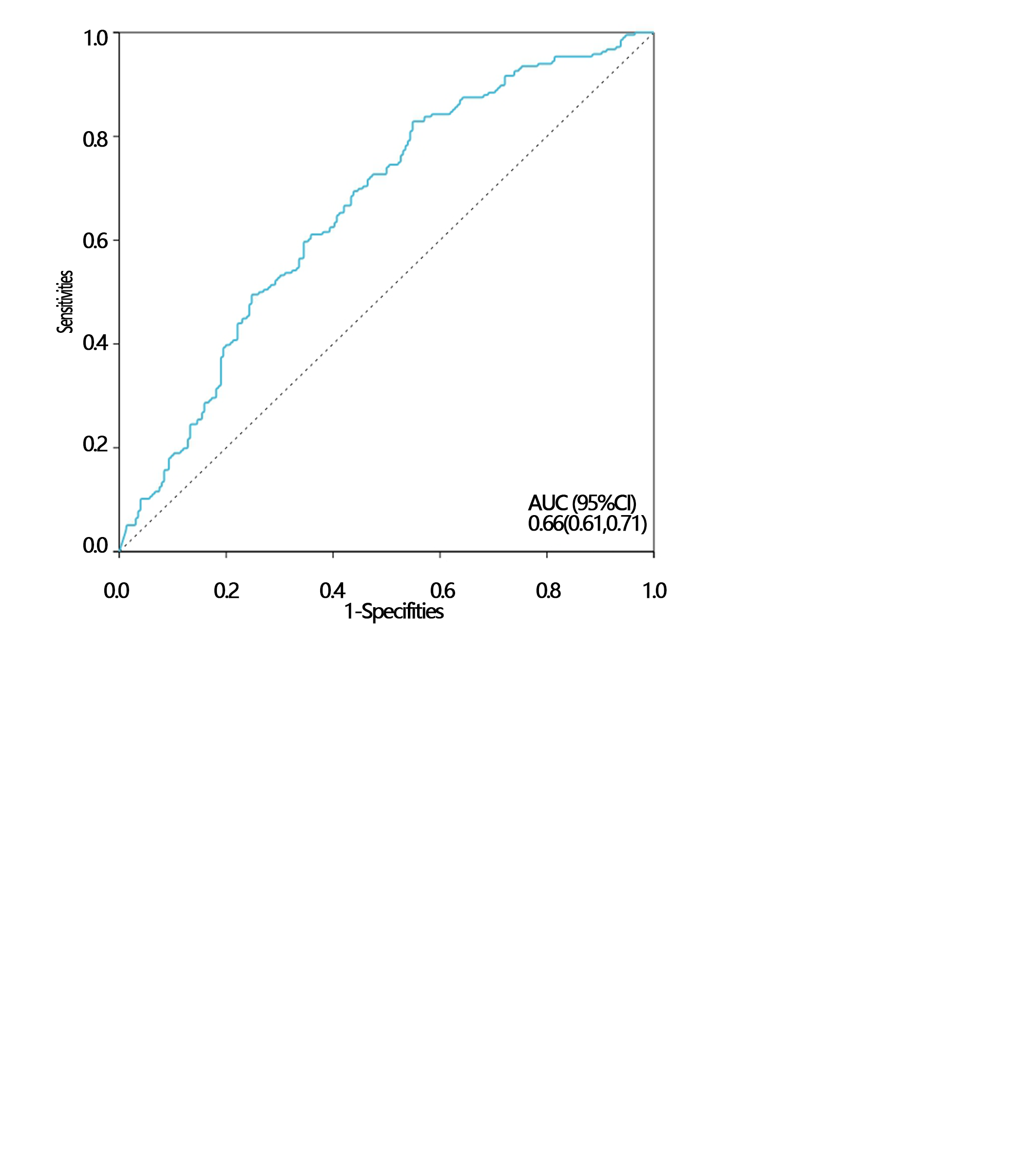

Supplement: Supplementary file 2 [file Image1.tif]
